# Trogocytosis-mediated expression of HER2 on immune cells may be associated with a pathological complete response to trastuzumab-based primary systemic therapy in HER2-overexpressing breast cancer patients

**DOI:** 10.1186/s12885-015-1041-3

**Published:** 2015-02-06

**Authors:** Eiji Suzuki, Tatsuki R Kataoka, Masahiro Hirata, Kosuke Kawaguchi, Mariko Nishie, Hironori Haga, Masakazu Toi

**Affiliations:** 1 Department of Breast Surgery, Kyoto University Hospital, 54 Shogoin kawahara-cho, Sakyo-ku, Kyoto, 606-8507 Japan; 2 Department of Diagnostic Pathology, Kyoto University Hospital, 54 Shogoin kawahara-cho, Sakyo-ku, Kyoto, 606-8507 Japan

**Keywords:** Breast cancer, HER2, Trogocytosis, Trastuzumab

## Abstract

**Background:**

Trogocytosis is defined as the transfer of cell-surface membrane proteins and membrane patches from one cell to another through contact. It is reported that human epidermal growth factor receptor 2 (HER2) could be transferred from cancer cells to monocytes via trogocytosis; however, the clinical significance of this is unknown. The aim of this study is to demonstrate the presence and evaluate the clinical significance of HER2^+^ tumor-infiltrated immune cells (arising through HER2 trogocytosis) in HER2-overexpressing (HER2+) breast cancer patients receiving trastuzumab-based primary systemic therapy (PST).

**Methods:**

To assess the trogocytosis of HER2 from cancer cells to immune cells, and to evaluate the up- and down-regulation of HER2 on immune and cancer cells, peripheral blood mononuclear cells from healthy volunteers and breast cancer patients were co-cultured with HER2+ and HER2-negative breast cancer cell lines with and without trastuzumab, respectively. The correlation between HER2 expression on tumor-infiltrated immune cells and a pathological complete response (pCR) in HER2+ breast cancer patients treated with trastuzumab-based PST was analyzed.

**Results:**

HER2 was transferred from HER2+ breast cancer cells to monocytes and natural killer cells by trogocytosis. Trastuzumab-mediated trogocytosed-HER2^+^ effector cells exhibited greater CD107a expression than non-HER2-trogocytosed effector cells. In breast cancer patients, HER2 expression on tumor-infiltrated immune cells in treatment naïve HER2+ tumors was associated with a pCR to trastuzumab-based PST.

**Conclusions:**

HER2-trogocytosis is visible evidence of tumor microenvironment interaction between cancer cells and immune cells. Given that effective contact between these cells is critical for immune destruction of target cancer cells, this interaction is of great significance. It is possible that HER2 trogocytosis could be used as a predictive biomarker for trastuzumab-based PST efficacy in HER2^+^ breast cancer patients.

**Electronic supplementary material:**

The online version of this article (doi:10.1186/s12885-015-1041-3) contains supplementary material, which is available to authorized users.

## Background

Human epidermal growth factor receptor 2 positive (HER2+) breast cancer cells are recognized by trastuzumab and undergo opsonization, which results in cell death by antibody-dependent cellular cytotoxicity (ADCC) in the presence of peripheral blood mononuclear cells (PBMCs). Following cancer cell–immune cell contact, an immune complex (IC) consisting of HER2, trastuzumab, and the Fcγreceptor (FcγR) of an effector cell, such as a natural killer (NK) cell or monocyte, is formed. The IC and small membrane fragments of the target cell, which surround the IC, are then transferred to the effector cell, resulting in reduced HER2 expression on the target cell surface. This phenomenon is broadly defined as trogocytosis [1], although the original definition of trogocytosis, as reported by Griffin et al., referred to the transfer of IC caps from the surface of lymphocytes to macrophages. This was mediated by macrophage Fc receptors [2], and occurred without destruction of the lymphocyte.

Although the overexpression and amplification of HER2 in breast cancer is associated with a poor prognosis, trastuzumab has provided clear clinical benefits in the primary systemic therapy (PST), adjuvant therapy, and metastatic breast cancer settings [3-5]. However, the majority of metastatic disease patients who do initially respond to trastuzumab generally acquire resistance within 1 year, and 20% of patients who receive trastuzumab in the adjuvant setting relapse. It is therefore necessary to elucidate the mechanisms responsible for treatment sensitivity and resistance. *In vitro* studies have indicated that trastuzumab has multiple mechanisms of action. Studies have shown that FcγR2A-131 polymorphisms impact a patient’s pathological response and can enhance the anti-tumor activity of trastuzumab, which is due, at least in part, to ADCC [6]. ADCC has been reported to occur in HER2+ breast cancer patients treated with trastuzumab. We believe that it may be possible to predict the efficacy of trastuzumab-based treatment of HER2+ breast cancer patients if the likelihood of ADCC can be determined. It is thought that cell–cell contact is necessary to induce ADCC by trogocytosis, and thus trogocytosis provides a potential mechanism to trace immune–cancer cell contact. We hypothesize that patients who show a greater degree of trogocytosis will exhibit a higher degree of ADCC.

Herein, we report that immune effector cells, such as CD14^+^ and CD56^+^ cells, express HER2 via trastuzumab-mediated trogocytosis. Furthermore, these trogocytosed-HER2^+^ immune effector cells show significantly higher levels of CD107a expression, a marker of target cancer cell cytotoxicity, compared to non-trogocytosed-HER2 immune effector cells. Importantly, we have found that in HER2+ breast cancer patients, trogocytosis can occur in the tumor microenvironment (TME) in the absence of trastuzumab. From this, we have hypothesized that patients who show a higher degree of HER2 trogocytosis prior to trastuzumab administration might show a better response to trastuzumab treatment; trastuzumab targeting of HER2+ tumor cells in these patients could be more effective and result in greater immune cell ADCC. Notably, we have found that patients who show a high degree of HER2 expression on tumor-infiltrated immune cells (by HER2 trogocytosis) demonstrate a significantly greater probability of achieving a pathological complete response (pCR) with trastuzumab-based PST. Thus, our data indicate that HER2 trogocytosis could be a predictive biomarker for the efficacy of trastuzumab-based PST in HER2+ breast cancer patients.

## Methods

### Cells

Her2/Neu-positive (HER2+) BT-474 and SK-BR-3 cell lines and Her2/Neu-negative (HER2^−^) MCF7 and MDA-MB-231 cell lines were obtained from the American Type Culture Collection. SK-BR-3, MDA-MB-231, and MCF7 cells were all cultured in RPMI 1640 containing 10% FBS, 100 U/mL penicillin, and 100 μg/mL streptomycin (Invitrogen). BT-474 cells were cultured in DMEM containing 10% FBS, 100 U/mL penicillin, and 100 μg/mL streptomycin. Cell lines were regularly tested and maintained negative for mycoplasma species. PBMCs were obtained from patients as part of their routine investigations at the Kyoto University Hospital. PBMCs were also obtained from healthy volunteers. Briefly, 8 mL of blood was collected using a VACUTAINER®CPT™ (Cell Preparation Tube; BD, Franklin Lakes, NJ). CPTs were stored at room temperature and processed in accordance with the manufacturer’s instructions within 6 h to collect the PBMCs and plasma. CD14^+^ monocytes and CD56^+^ NK cells were isolated by depletion (negative selection) of non-monocyte and non-NK cells, respectively, according to the manufacturer’s instructions (Pan Monocyte Isolation Kit (Cat. No. 130-096-537) and NK cell isolation Kit (Cat. No. 130-092-657), Miltenyi Biotec). The isolated PBMCs, monocytes, and NK cells were used in assays immediately.

### Tumor dissociation

Immediately after surgical resection, solid breast tumor samples were minced and dissociated into single-cell suspensions by incubating at 37°C for 1 h with 1 M HEPES cell dissociation buffer containing 200 U/mL of Liberase TM (Roche) in basic accordance with the modified protocol reported by Panchision et al. [7]. This method was evaluated and found to yield a cell suspension with appropriate dissociation efficiency, cell viability, and antigen retention for analysis by flow cytometry.

### Trogocytosis assay

Isolated PBMCs, monocytes, and NK cells (effector cells) were co-cultured with different ratios (1:1, 10:1, 100:1, or 1000:1) of human breast cancer cell lines or breast cancer patient tumor cells (target cells) in RPMI 1640 alone or in RPMI 1640 containing different concentrations of normal human plasma. Cells were co-cultured in Eppendorf 500 Tubes® (C153008O, Eppendorf AG, Hamburg, Germany) for 60 min at 37°C, 5% CO_2_, in the presence of different concentrations of trastuzumab (0, 0.1, and 1 μg/mL; provided by Chugai Pharmaceutical Co., Ltd.). The optimal time and effector:target (E:T) cell ratios were determined in preliminary studies (data not shown). After co-culture, the cells were transferred to fluorescence-activated cell sorter tubes (Corning, Cat. No. 352235), washed with 0.5% BSA-PBS, and centrifuged at 300 × *g* for 5 min. The supernatant was discarded and the cells were re-suspended in 0.5% BSA-PBS and analyzed by flow cytometry using a FACSCalibur (BD Biosciences). The expression of HER2 (stained with FITC, PE, or APC-conjugated anti-HER2 antibodies; BD Biosciences) was determined on target breast cancer cells and on CD14^+^ (stained with FITC-conjugated anti-CD14 antibodies; BD Biosciences, Cat. No. 555397), CD56^+^ (stained with PE-conjugated anti-CD56 antibodies; BD Biosciences, Cat. No. 555516), CD19^+^ (stained with FITC-conjugated anti-CD19 antibodies; BD Biosciences, Cat. No. 557398), and CD3^+^ (stained with PE-conjugated anti-CD3 antibodies; BD Biosciences, Cat. No. 555340) cells. Antibodies were diluted 1:20 with Flow Cytometry Staining Buffer (BD Biosciences, Cat. No. 00-4222-26) prior to staining. Positive cell populations were gated with reference to negative isotype control matched antibody staining reactions (Mouse IgG1 PE and APC, Cat. No. 559320 and 554681, respectively; Mouse IgG2a FITC, Cat. No. 553456; BD Biosciences) and baseline HER2 expression on CD14^+^, CD56^+^, CD19^+^, and CD3^+^ cells from normal healthy volunteer PBMCs (without cancer cell co-culture). Data were stored electronically for reanalysis (FlowJo Version 7.6.5 software; TreeStar).

### ADCC assay

A mixture of effector and breast cancer cells (E:T ratio = 10:1, the same ratio as the trogocytosis assay) was prepared in RPMI 1640 medium and treated with trastuzumab (0, 0.1, or 1 μg/mL) for 90 min at 37°C, 5% CO_2,_ prior to performing the CD107a (PE-Cy5-conjugated anti-CD107a antibody; eBioscience, San Diego, CA) degranulation assay.

### Staining of tumor specimens

Surgical specimens from HER2+ breast cancer patients were paraffin embedded and 4 μm sections were cut. After deparaffinization with xylene, tissue sections were rehydrated and endogenous peroxidase activity was quenched with 3% hydrogen peroxide for 10 min. After steaming for 20–30 min using an electric pressure cooker (SR-P37, Panasonic, Tokyo, Japan), the sections were blocked with 5% normal goat serum (Abcam), and incubated with both anti-human CD14 (Diluted in 1:50, Clone 7, mouse monoclonal; Leica) and anti-HER2 (Ready-to-Use, Clone 4B5, rabbit monoclonal; Roche) primary antibodies for 1 h. Alexa Fluor 488-conjugated anti-mouse IgG (diluted in 1:200, ab150117; Abcam) and Alexa Fluor 594-conjugated anti-rabbit IgG (diluted in 1:200, ab150084; Abcam) secondary antibodies were used for immunofluorescence staining of CD14 and HER2, respectively. The secondary antibodies were applied for 1 h prior to mounting with Fluoroshield mounting medium with DAPI (Abcam). To evaluate the correlation between HER2-trogocytosis and a pCR, sections were stained using MACH2 double stain 2 (Biocare Medical) for 1 h, followed by Vulcan Fast Red (Biocare Medical) addition for HER2 staining.

### Statistical analysis

Statistical analyses, including the Student’s *t* test, Wilcoxon signed-rank test, and Chi-square test were performed using JMP Pro 11.

### Ethical considerations

In accordance with the Declaration of Helsinki, informed consent was obtained from all breast cancer patients and healthy volunteers. The study was approved by the institutional ethics review committee of Kyoto University Hospital (Protocol G424).

## Results

### Trogocytosis is specifically observed in HER2+ breast cancer cell lines and CD14^+^ cells exhibited a greater degree of trogocytosis than CD56^+^ cells in *in vitro* trogocytosis assays

**Figure 1:**
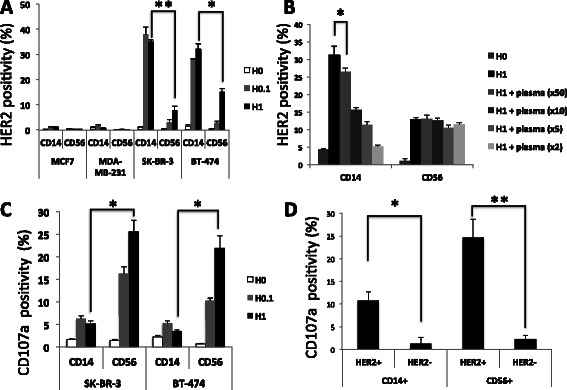
**HER2-trogocytosis and CD107a expression on immune effector cells in human breast cancer cell lines.** HER2+ SK-BR-3 and BT-474 cell lines and HER2^−^ MCF7 and MDA-MB-231 cell lines were used as target cells, and healthy human PBMCs were used as effectors. Cells were co-cultured for 60 min in the trogocytosis assay and 90 min in the CD107a degranulation assay. Cells were stained with FITC-CD14, PE-CD56, APC-HER2, and PE-Cy5-CD107a antibodies and subjected to flow cytometry. **A** The trogocytosis assay was performed with an effector:target (E:T) cell ratio of 10:1 and various concentrations of trastuzumab (H: H0, without trastuzumab; H0.1, 0.1 μg/mL of trastuzumab; H1, 1 μg/mL of trastuzumab). *P < 0.05; **P < 0.001. **B** The trogocytosis assay was performed with an E:T cell ratio of 10:1 and 1 μg/mL of trastuzumab (H1). Normal human plasma was added to the co-culture medium at various dilutions (1:50, 1:10, 1:5, and 1:2). The target cancer cells used in the assay were SK-BR-3. *P < 0.05. **C** The antibody-dependent cellular cytotoxicity (ADCC) assay was performed with an E:T cell ratio of 10:1 and various concentrations of trastuzumab (H). CD107a positivity is indicative of CD14^+^ and CD56^+^ cell target cancer cell cytotoxicity. *P < 0.05. **D** CD107a positivity on HER2^+^/CD14^+^, HER2^−^/CD14^+^, HER2^+^/CD56^+^, and HER2^−^/CD56^+^ cells is shown. The target cancer cells used in the assay were SK-BR-3. *P < 0.05; **P < 0.001. HER2 positivity represents the uptake of HER2 onto CD14^+^ and CD56^+^ effector cells. All figures show the mean ± SEM. Experiments were performed for 3 healthy volunteers at least 2 times and similar data were obtained each time. All figures show the results from a single representative experiment.

Initially, we performed a trogocytosis assay to determine whether trastuzumab-mediated trogocytosis specifically occurred in HER2+ breast cancer cells. We used the HER2+ SK-BR-3 and BT-474 and HER2^−^ MCF7 and MDA-MB-231 breast cancer cell lines. An E:T cell ratio of 10:1 was used, and 0, 0.1, or 1 μg/mL of trastuzumab (H) was added for 60 min. The E:T cell suspensions were stained with FITC-CD14, PE-CD56, and APC-HER2 antibodies and were analyzed by flow cytometry. In the cell suspensions with HER2+ target cells, both the CD14^+^ and CD56^+^ cells expressed HER2 on their cell surface, indicative of HER2-trogocytosis. The proportion of CD14^+^ cells that were also HER2^+^ was significantly higher in the HER2+ SK-BR-3 and BT-474 cell suspensions treated with 0.1 and 1 μg/mL of trastuzumab than in the HER2^−^ MCF7 and MDA-MB-231 cell suspensions treated in the same way. Furthermore, the CD14^+^ cells showed significantly more HER2-trogocytosis than the CD56^+^ cells in both the SK-BR-3 and BT-474 cell suspensions (Figure 1A). In order to determine whether IgG1 antibodies present in normal human plasma affect HER2 trogocytosis, we performed the trogocytosis assay in RPMI 1640 medium supplemented with different dilutions of normal human plasma (1:2, 1:5, 1:10, and 1:50). The results indicated that the CD14^+^ cell HER2-trogocytosis observed in Figure 1A was abrogated in a normal human plasma dose-dependent manner (Figure 1B). We proceeded to investigate whether effector cells, such as CD19^+^ B cells and CD3^+^ T cells, other than CD14^+^ monocytes and CD56^+^ NK cells, also demonstrated HER2 trogocytosis. However, no increased HER2 expression was observed on CD19^+^ or CD3^+^ cells in the trogocytosis assay (Additional file 1: Figure S1A).

### CD107a is predominantly expressed on CD56^+^ rather than CD14^+^ cells in 90 min ADCC assays

Having shown that normal human plasma potentially inhibits CD14^+^ cell HER2-trogocytosis, we eliminated plasma from the medium and buffer of further experiments. This was particularly important given that the clinical relevance and mechanisms of this plasma inhibitory effect are not fully understood.

We proceeded to measure the target cell cytotoxicity of effector cells by quantifying the extracellular expression of the degranulation marker CD107a on CD14^+^ and CD56^+^ cells. SK-BR-3 and BT-474 cells were used as target cells and the ADCC assay was performed as specified in the Methods section. CD107a expression on CD14^+^ and CD56^+^ cells began to be observed after 60 min in the ADCC assay and reached a plateau after 90 min. We therefore performed a 90 min ADCC assay to quantify CD107a expression. Although the mechanism of target cell killing is different between CD14^+^ and CD56^+^ cells, both the CD14^+^ and CD56^+^ cells expressed CD107a in a trastuzumab dose-dependent manner. When the level of CD107a expression was analyzed on total CD14^+^ or CD56^+^ cells, CD107a expression was significantly greater on CD56^+^ cells than on CD14^+^ cells (Figure 1C).

### Immune effector cells that express HER2 (by HER2-trogocytosis) express significantly higher levels of CD107a

To confirm the role of trogocytosis in ADCC, the level of CD107a expression on trogocytosed-HER2^+^/CD14^+^ and trogocytosed-HER2^+^/CD56^+^ cells was compared to CD107a expression on non-HER2-trogocytosed HER2^−^/CD14^+^ and HER2^−^/CD56^+^ cells. Using flow cytometry, it was shown that CD107a expression was significantly higher on the trogocytosed-HER2^+^/CD14^+^ and trogocytosed-HER2^+^/CD56^+^ cells than the non-HER2-trogocytosed immune effector cells (Figure 1D). Representative dot plots are shown in Additional file 1: Figure S1B.

### Trogocytosis reduces HER2 expression on HER2+ target cancer cells and HER2 expression on isolated CD14^+^ and CD56^+^ immune cells in the absence of trastuzumab was also shown

**Figure 2:**
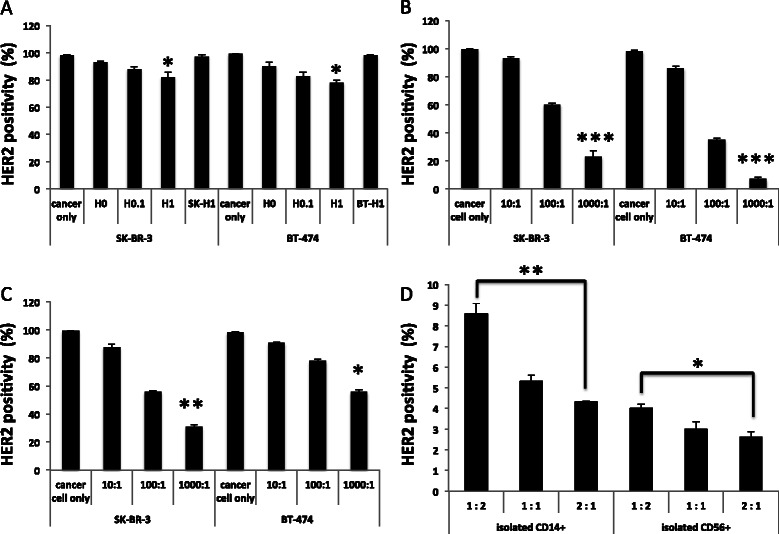
**Reduction in HER2 expression in HER2**^**+**^**target human breast cancer cell lines and HER2 expression on isolated CD14**^**+**^**and CD56**^**+**^**immune cells in the absence of trastuzumab.** SK-BR-3 and BT-474 were used as targets and healthy human PBMCs were used as effectors. For the trogocytosis assay, cells were co-cultured for 60 min. Cell mixtures were subsequently stained with an APC-HER2 antibody and subjected to flow cytometry. **A** The trogocytosis assay was performed with an E:T cell ratio of 10:1 and various concentrations of trastuzumab (H: H0, without trastuzumab; H0.1, 0.1 μg/mL of trastuzumab; H1, 1 μg/mL of trastuzumab). SK-H1 and BT-H1; SK-BR-3 and BT-474 cells treated with 1 μg/mL of trastuzumab alone, respectively. **B** The trogocytosis assay was performed with an E:T cell ratio of 10:1, 100:1, and 1000:1 and 1 μg/mL of trastuzumab. **C** The trogocytosis assay was performed with an E:T cell ratio of 10:1, 100:1, and 1000:1 without trastuzumab treatment. *P < 0.05; **P < 0.01; ***P < 0.001 versus cancer cells only. **D** The trogocytosis assay was performed with isolated CD14^+^ or CD56^+^ cells in 1:2, 1:1 and 2:1 ratio with SK-BR-3 cells and without trastuzumab (H0). The level of HER2 expression on the CD14^+^ and CD56^+^ cells is shown as the percent of HER2^+^ cells in each condition. *P < 0.05; **P < 0.001. All figures show the mean ± SEM. Experiments were performed for a single healthy volunteer twice and similar data were obtained each time.

The results from the trogocytosis assay indicated that HER2 expression on target cancer cells was reduced as the trastuzumab dose increased (Figure 2A). However, HER2 expression in SK-BR-3 and BT-474 cells does not decrease when they were incubated with trastuzumab alone for 60 min (Figure 2A: SK-H1 and BT-H1, respectively). This result suggests that 60 min is insufficient for direct internalization of the HER2-trastuzumab complex, and that trogocytosis is the key mechanism responsible for the loss of HER2 in the 60 min trogocytosis assay. We further confirmed that with higher E:T ratios, a greater decrease in HER2 expression was observed in both the trastuzumab-dependent (TD; Figure 2B) and trastuzumab-independent (TI; Figure 2C) trogocytosis assays. To confirm that the uptake of HER2 onto CD14^+^ and CD56^+^ effector cells in TI trogocytosis, we assayed TI trogocytosis using purified CD14^+^ and CD56^+^ cells with E:T cell ratios of 1:2, 1:1 and 2:1. Although the precise mechanisms of TI HER2-trogocytosis are not clear, by performing trogocytosis assays in trastuzumab-free conditions, we found that CD14^+^ and CD56^+^ cells in CD14^+^ and CD56^+^ cells purified from PBMCs (91.5% and 96.9% purity, respectively; data not shown) express HER2 on the cell surface by trogocytosis. As the T:E cell ratio increased, the quantity of HER2 on the surface of isolated CD14^+^ cells and CD56^+^ cells also increased (Figure 2D).

### Patient PBMCs exhibit HER2-trogocytosis although the extent is variable

**Figure 3:**
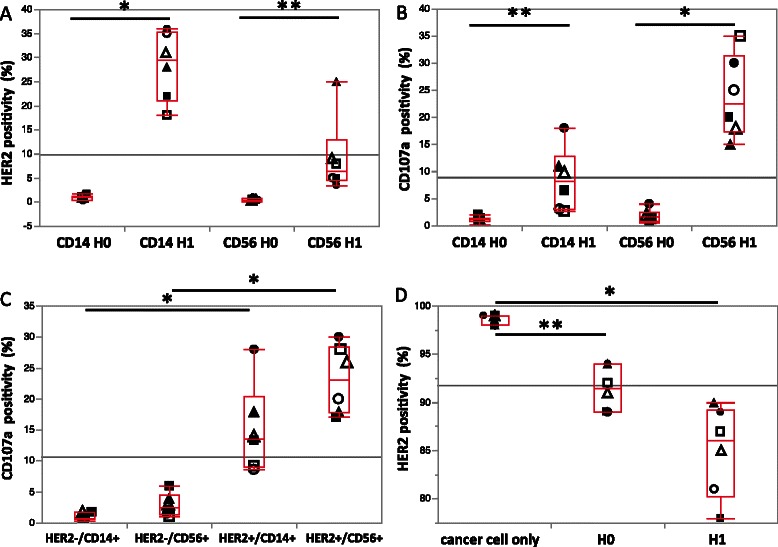
**ADCC and trogocytosis in PBMCs from HER2+ breast cancer patients and healthy volunteers.** SK-BR-3 cells were used as target cells in all experiments and PBMCs from HER2+ breast cancer patients (N = 3) and healthy volunteers (N = 3) were used as effector cells. The SK-BR-3 cells and PBMCs were co-cultured for 60 min in the trogocytosis assay and 90 min in the CD107a degranulation assay. Cell mixtures from the assays were stained with FITC-CD14, PE-CD56, APC-HER2, and PE-Cy5-CD107a antibodies and subjected to flow cytometry. **A** The trogocytosis assay was performed with an E:T cell ratio of 10:1 with and without trastuzumab (H0, without trastuzumab; H1, 1 μg/mL of trastuzumab). HER2 positivity represents the uptake of HER2 onto CD14^+^ and CD56^+^ effector cells. **B** The ADCC assay was performed with an E:T cell ratio of 10:1 with and without trastuzumab (H0, without trastuzumab; H1, 1 μg/mL of trastuzumab). CD107a positivity is indicative of target cancer cell cytotoxicity by CD14^+^ and CD56^+^ cells. **C** CD107a positivity on HER2^+^/CD14^+^, HER2^−^/CD14^+^, HER2^+^/CD56^+^, and HER2^−^/CD56^+^ cells is shown. **D** The trogocytosis assay was performed with an E:T cell ratio of 10:1 with and without trastuzumab (H0, without trastuzumab; H1, 1 μg/mL of trastuzumab). The level of HER2 expression on the trogocytosed SK-BR-3 cells is shown as the percent of HER2^+^ cells in each condition. *P < 0.005, **P < 0.05. All figures show the mean ± SD. Healthy volunteers are represented by open circles, squares, and triangles; patients are represented by closed circles, squares, and triangles.

To investigate the clinical significance of our findings, we examined the trogocytosis potential of freshly isolated PBMCs from early stage HER2+ breast cancer patients. Similar to the PBMCs of healthy volunteers, both the CD14^+^ and CD56^+^ immune effector cells in the PBMCs of breast cancer patients’ showed HER2-trogocytosis (Figure 3A) and target cell cytotoxicity (Figure 3B). Furthermore, the trogocytosed-HER2^+^/CD14^+^ and trogocytosed-HER2^+^/CD56^+^ cells from patients’ showed significantly higher CD107a expression (Figure 3C). A reciprocal reduction in HER2 expression on the HER2+ cancer cells was also observed in the patient’s cells (Figure 3D). However, there was also a large variation in the degree of trogocytosis and the extent of HER2 reduction among patients and healthy volunteers. Although the increased HER2 expression on CD14^+^ or CD56^+^ cells was not robustly correlated with reduced HER2 cancer cell expression, the diversity in response suggests that trogocytosis has the potential to be used as a predictive marker for trastuzumab-based treatment efficacy in breast cancer patients.

### Flow cytometry of HER2+ breast cancer patient’s tumor cells indicates high HER2 expression on CD14^+^ and CD56^+^ cells

In order to confirm that HER2 was expressed on tumor-infiltrated immune cells, and determine whether HER2 could be transferred to immune effector cells by trogocytosis, we isolated individual tumor cells from a HER2+ breast cancer patient treated with trastuzumab (N = 1) and a HER2^−^ luminal type breast cancer patient. Dissociated tumor tissue cell suspensions were subjected to flow cytometry and forward/side scatter, CD14, and CD56 staining was used to distinguish the cancer cell, monocyte, and lymphocyte populations. Cancer cells and monocytes were of a similar size but monocytes were CD14^+^ (Additional file 2: Figure S2; cancer cells indicated by red circle and monocytes indicated by yellow circle). Lymphocytes were smaller than cancer cells and monocytes and appeared as a distinct cell population (Additional file 2: Figure S2; indicated by blue circle). Interestingly, flow cytometry indicated that HER2 expression was higher on the patient’s tumor-infiltrated CD14^+^ cells than the CD14^+^ PBMCs (19.3% and 1.29%, respectively; Additional file 2: Figure S2A). As a negative control, we also tested HER2 expression on the CD14^+^ cells of a HER2^−^ luminal type breast cancer patient; no HER2 expression was observed on the CD14^+^ cells of the luminal type breast cancer patient (Additional file 2: Figure S2A; luminal type tumor). The CD56^+^ NK cells from tumors were identified by CD56^+^ staining in the lymphocyte population (identified through their forward/side scatter properties), which had previously been identified as being smaller than the cancer cells and monocytes (red and green circles, respectively; Additional file 2: Figure S2B). Similar to the CD14^+^ immune effector cells, the tumor-infiltrated CD56^+^ cells from HER2+ breast cancer patients expressed high levels of HER2 and the CD56^+^ cells from luminal type breast cancer patient showed little HER2 expression (Additional file 1: Figure S2B).

### HER2 can be transferred from tumor cells to CD14^+^ and CD56^+^ immune cells by autologous trogocytosis

A single autologous trogocytosis assay was performed by co-culturing digested tumor cell suspensions and PBMCs from the HER2+ patient at a ratio of 1:10 with either 0 or 1 μg/mL of trastuzumab. The number of HER2^+^/CD14^+^ and HER2^+^/CD56^+^ cells was higher in the co-culture treated with 1 μg/mL of trastuzumab than in the untreated co-culture (Additional file 3: Figure S3A, CD56^+^ and CD14^+^ cells). Furthermore, HER2 expression on digested tumor cells was lower in co-cultures treated with 1 μg/mL of trastuzumab than in cultures treated with 0 μg/mL of trastuzumab (18% and 25%, respectively; Additional file 3: Figure S3A, Tumor cell). Although direct HER2 internalization by trastuzumab is one possible mechanism of down-modulation of HER2, these findings suggest that HER2 can be transferred from HER2+ breast tumor cells to CD14^+^ and CD56^+^ cells, which provides potential evidence for trogocytosis within the TME of HER2+ breast cancer patients.

### Confirmation of TI trogocytosis using autologous trogocytosis assays

To investigate the potential for TI trogocytosis, digested tumor cell suspensions and autologous PBMCs from the HER2+ patient were co-cultured in various ratios without trastuzumab; HER2 expression reduced as the E:T cell ratio increased (Additional file 3: Figure S3B). This suggests that cancer cell–immune cell contact occurs in the absence of a HER2-targeting antibody, and this could also occur in the TME of HER2+ breast cancer patients. Such contact could result in immune cells that express trogocytosed-HER2 after encountering HER2 expressing cancer cells. This is clinically significant because it may enable the efficacy of trastuzumab-based treatment to be predicted in individual HER2+ breast cancer patients by evaluating the probability of immunological HER2-trogocytosis.

### Tumor-infiltrated immune cell HER2 expression may be associated with a pCR

**Figure 4:**
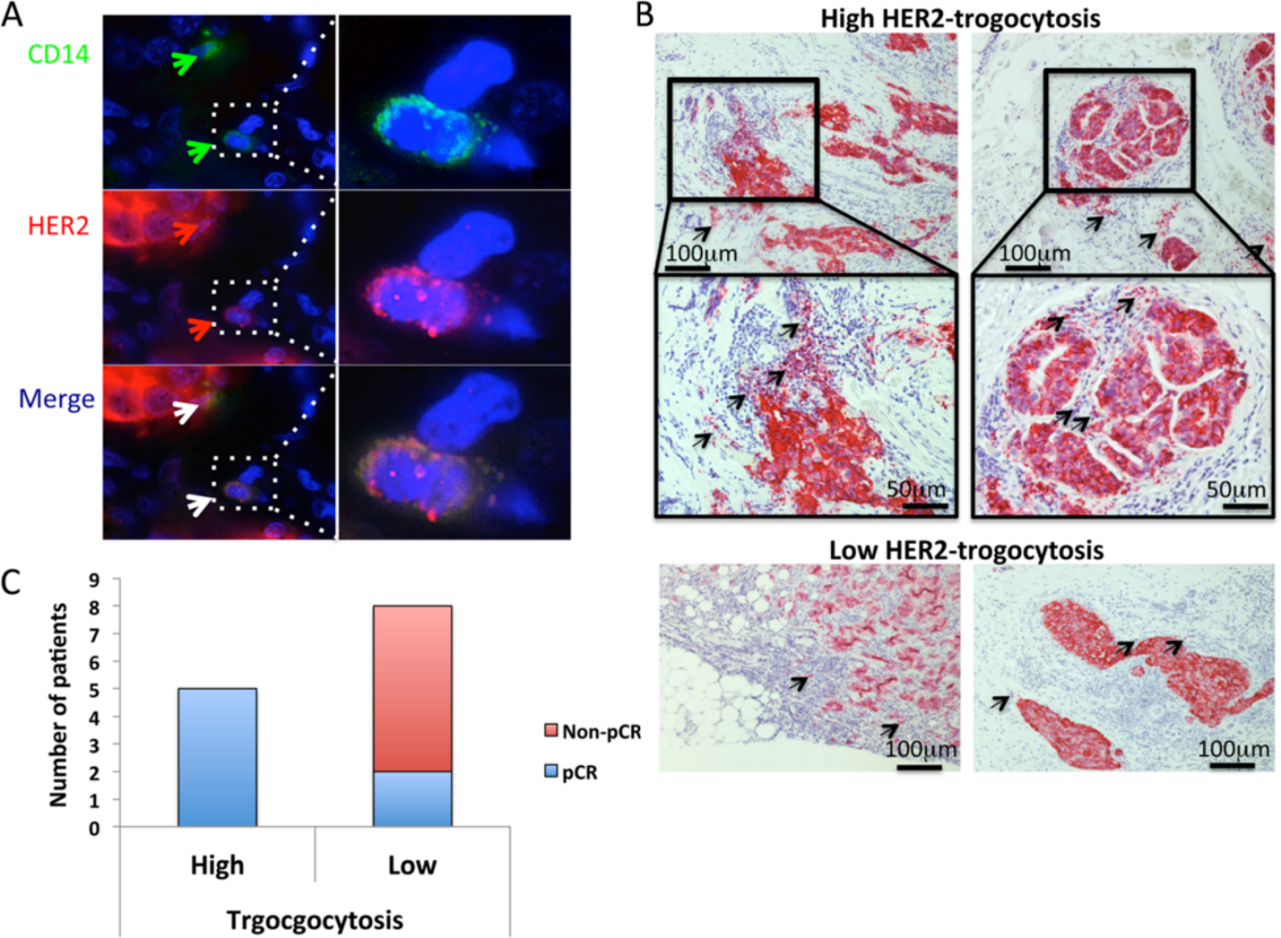
**HER2 expression on the tumor infiltrated immune cells of HER2+ breast cancer patients. A** Representative immunofluorescence staining of CD14^+^/HER2^+^ cells (HER2-trogocytosis) in HER2+ breast cancer tissues. Green arrow, CD14^+^ cells; red arrow, HER2^+^ cells; white arrow, CD14^+^/HER2^+^ cells. **B** Representative immunohistochemical staining of HER2 in HER2+ breast cancer tissues before systemic treatment. Black arrows indicate HER2^+^ tumor infiltrated immune cells. **C** Correlation between HER2-trogocytosis and a pathological complete response (pCR) in 13 HER2+ breast cancer patients treated with trastuzumab-based primary systemic therapy (*P* < 0.05).

To investigate whether HER2-trogocytosis prior to treatment could be used to predict HER2+ breast cancer patients’ responses to PST consisting of anthracyclin followed by taxan plus trastuzumab, we evaluated HER2 trogocytosis in formalin-fixed paraffin embedded tumor samples collected from patients at Kyoto University Hospital from 2008 to 2012 (N = 13; 7 pCR and 6 non-pCR patients were included). The patient’s clinicopathological information is shown in Table 1. HER2^+^ tumor-infiltrated immune cells (trogocytosed-HER2^+^ immune cells) were analyzed by immunohistochemical staining, which was interpreted by an expert pathologist who was blind to patient information. The absolute number of trogocytosed-HER2^+^ immune cells in peri-tumor area hotspots was counted; the median was 11. We defined a highly trogocytosed tumor as one in which 12 or more trogocytosed-HER2^+^ immune cells were present and a lowly trogocytosed tumor as one in which fewer than 11 trogocytosed-HER2^+^ immune cells were present. Representative immunofluorescence and immunohistochemical staining of HER2^+^ tumor-infiltrated immune cells in HER2+ breast cancer tissues are shown in Figures 4A and B. The correlation between trogocytosed-HER2^+^ immune cells and patient response was analyzed; we found that patients with a high degree of HER2-trogocytosis had a significantly greater probability of achieving a pCR with PST consisting of 3–4 courses of FEC100 followed by 12 courses of paclitaxel and trastuzumab than patients with a low level of HER2-trogocytosis (P = 0.023; Figure 4C).

**Table 1:** **Patient clinicopathological information**

Sample ID	Treatment	Response	ER (%)	PgR (%)	HE R2 (IHC)	Ki67 (%)
07-9749	FEC → PH	non pCR	0	0	3+	NA
08-9679	FEC → PH	non pCR	100	80	3+	30
09-3788	FEC → PH	pCR	0	0	3+	10
09-5807	FEC → PH	pCR	80	30	3+	30
10-248	FEC → DH	pCR	60	70	3+	25
10-7977	FEC → PH	non pCR	30	0	3+	20
10-8712	FEC → PH	pCR	0	1	3+	40
11-577	FEC → PH	non pCR	5	0	3+	30
11-5192	FEC → PH	non pCR	10	30	3+	80
11-10011	FEC → PH	pCR	100	40	3+	22
12-2401	FEC → PH	pCR	10	0	3+	39.6
10-09897	FEC → PH	pCR	0	0	3+	25
10-06979	FEC → PH	non pCR	0	0	3+	10

FEC, 5-fluorouracil epirubicin cyclophosphamide; P, paclitaxel; D, docetaxel; H, trastuzumab.

## Discussion

*In vitro* trastuzumab-mediated trogocytosis has been extensively investigated by Beum et al., who have shown that trogocytosis of monoclonal antibody (trastuzumab, rituximab, or cetuximab)-opsonized cells is mediated by PBMC, THP-1, and primary monocytes. It is likely that these monoclonal antibodies, and potentially other anti-cancer monoclonal antibodies now used in the clinic, also promote trogocytic removal of the therapeutic antibody and their cognate antigens from tumor cells *in vivo* [1]. In the present study, we aimed to demonstrate the existence and clinical significance of HER2^+^ tumor-infiltrated immune cells in HER2+ breast cancer patients receiving trastuzumab treatment. To the best of our knowledge, this is the first study to report potential evidence for HER2 transfer from HER2+ breast cancer cells to immune cells, including CD14^+^ and CD56^+^ cells, by trogocytosis in HER2+ breast cancer patients (Additional file 2: Figure S2 and Figure 4). Moreover, the presented findings could provide a novel strategy, beyond the conventional evaluation of HER2 expression, for predicting the patients who are most likely to achieve a pCR with preoperative trastuzumab-based systemic therapy; it may be possible to identify patients who are likely to achieve a pCR by evaluating the status of HER2 expression on tumor-infiltrated immune cells (Figure 4). Importantly, as shown in Figure 1B, CD14^+^ cell HER2-trogocytosis was abrogated in the presence of normal human plasma. Herceptin is an IgG1 kappa light chain antibody. Human serum/plasma contains a high concentration of IgG1 kappa light chain antibodies. PBMCs, which were used in the *in vitro* trogocytosis assays, can bind to IgG1 antibodies, such as trastuzumab or those stemming from human serum/plasma. As such, it is possible that trastuzumab and human serum/plasma IgG1 antibodies may compete with one another *in vivo* for binding to immune cell receptors, which could account for the inconsistent *in vitro* results. However, the clinical relevancy and precise mechanisms of this action remain unclear and the future experimental and clinical studies are required.

In order to confirm whether trogocytosis is caused by trastuzumab in HER2+ breast cancer patients, it would be necessary to compare the levels of HER2 expression on tumor-infiltrated immune cells before and after trastuzumab treatment. However, practically, it is difficult to analyze fresh tumor samples by flow cytometry prior to cancer diagnosis and identification of the biological phenotype, for example, the ER or HER2 status. As shown in Additional file 2: Figure S2, a significant increase in HER2 expression on tumor-infiltrated CD14^+^ and CD56^+^ cells was observed after administration of the anti-HER2 antibody trastuzumab to HER2+ breast cancer patients. Although it cannot be definitively concluded that HER2 was transferred from the cancer cells to the CD14^+^ and CD56^+^ cells by trogocytosis, the observation is significant because HER2 expression is not usually observed on CD14^+^ and CD56^+^ cells in normal PBMCs (Additional file 2: Figures S2A and B). Supporting the notion that HER2 trogocytosis is specific to HER2+ breast cancer, only low levels of HER2 expression were observed on the CD14^+^ and CD56^+^ cells of luminal type tumors (Additional file 2: Figures S2A and B; luminal type tumor). Moreover, a trastuzumab concentration-dependent attenuation of target cancer cell HER2 expression (although direct HER2 internalization by trastuzumab is one possible mechanism of down-modulation of HER2), and reciprocal increase in CD14^+^ and CD56^+^ immune cell HER2 expression, was observed in the HER2+ patient tumor cell–autologous PBMC trogocytosis assay (Additional file 3: Figure S3A). Although we had previously thought that trogocytosis could occur only allogeneically, this finding suggests that autologous transfer of HER2 from cancer cells to CD14^+^ and CD56^+^ cells might occur. Ross et al. have investigated trogocytosis in both the allogeneic experimental setting and in patients with multiple myeloma and other B-cell malignancies; they identified 2 molecules as potential transfer candidates, human leukocyte antigen (HLA)-G and the B7 molecule CD86, and identified T cells as the most common recipient lymphocyte subpopulation [8]. It was this evidence that encouraged us to conduct trogocytosis studies using human breast cancer cell lines and human PBMCs, even though these were only allogeneic-type experiments.

The question of whether HER2 expression on CD14^+^ monocytes following trogocytosis is effective at providing an acquired immune response against HER2+ breast cancer cells is an important one. The molecular phenotype of HER2 expression on CD14^+^ monocytes has not been clearly investigated and, as such, our understanding of the extent and clinical significance of this trogocytosis is still very limited [9-14]. However, there are several reports that suggest that trogocytosis might act to stimulate immunological tolerance or immune effector cell activation [8,15-18]. We performed trastuzumab-dependent (TD) and trastuzumab-independent (TI) trogocytosis assays using PBMCs from healthy volunteers and HER2+ breast cancer patients as effector cells and HER2+ breast cancer cell lines as the target cells. We found that the CD14^+^ immune effector cell subset showed greater TD HER2 trogocytosis than the CD56^+^ cells (Figures 1A and 3A). In addition, CD56^+^ cells showed greater CD107a expression than the CD14^+^ cells (Figures 1C and 3B). Little or no HER2 expression was observed on the CD14^+^ and CD56^+^ PBMCs of the healthy volunteers and HER2^−^ breast cancer patient; similarly, little or no HER2 expression was observed on the tumor-infiltrated CD14^+^ and CD56^+^ PBMCs of the HER2^−^ breast cancer patient. Thus, we believe that HER2 expression on CD14^+^ or CD56^+^ cells is indicative of contact between immune cells and HER2+ cancer cells. Tight cancer–immune cell contact is critical for target cancer cell destruction by immune effector cells [19] and, as such, the trogocytosed-HER2^+^ immune cells should exhibit effective trastuzumab-mediated target cancer cell ADCC. Indeed, the trogocytosed-HER2^+^ immune effector cells showed higher levels of CD107a expression than the non-HER2-trogocytosed immune effector cells (Figures 1D and 3C). We therefore conclude from the current study that HER2 trogocytosis is proof of target cancer cell elimination by ADCC.

In this study, TI and TD HER2 trogocytosis by immune effector cells was shown to result in a reduction in HER2 expression on target HER2+ breast cancer cells (Figure 2A-C). This finding indicates a possible role for HER2 trogocytosis in modulating HER2 expression on HER2+ breast cancer cells. However, most studies to date have indicated that loss of the HER2 extracellular domain (ECD) is principally caused by shedding of the HER2 ECD or direct internalization of the trastuzumab-HER2 complex. Despite lacking the majority of the ECD, truncated HER2 receptors have been shown to be capable of stimulating breast cancer progression *in vivo* and in clinical studies of breast cancer patients [20,21]. HER2 shedding plays an important role in trastuzumab treatment response and resistance. However, the interaction of immune cells with HER2+ trastuzumab-opsonized cancer cells in the TME is also a crucial factor in trastuzumab treatment response. We believe that TME HER2 trogocytosis by immune effector cells is an important mechanism of HER2 reduction, which could potentially affect trastuzumab treatment outcome. The trogosytosis assays, shown in Figure 2, indicate that HER2 expression on target cancer cells was down-regulated in both SK-BR-3 and BT-474 cells. Previous western blotting studies of cell lysates have indicated that full-sized transmembrane major histocompatibility complex (MHC) class I protein and cognate NK-cell receptor exchange can occur between cells, and that intact MHC class I protein can be transferred from antigen presenting cells to T cells [9]; this indicates that trogocytosis does not involve proteolytic cleavage. Furthermore, trogocytosed proteins can commonly be detected by monoclonal antibodies targeted against both extracellular epitopes and intracellular fluorescent protein tags, further indicating that both the intracellular and extracellular epitopes of transmembrane proteins are transferred [22,23]. This leads us to speculate that the full sized HER2, including the ECD and intracellular phosphorylation domain, is transferred during HER2-trogocytosis. This is distinct from proteolytic shedding of HER2, and consequently, we believe that HER2 trogocytosis may inhibit HER2 intracellular signal transduction, which could induce target cancer cell death. We therefore further hypothesize that TD HER2 trogocytosis, which results in reduced target cancer cell HER2 expression, could induce target cancer cell death by pro-apoptotic proteins, such as granzymes and TNF-alpha, in addition to inducing trastuzumab-mediated ADCC. As such, increased trogocytosis induction may be associated with improved trastuzumab treatment efficacy.

From the presented results, we believe that the TI HER2 trogocytosis results are of the greatest clinical significance. Patients who exhibited a greater degree of TI HER2 trogocytosis achieved a greater degree of HER2 trogocytosis following HER2+ breast cancer cell targeting by trastuzumab, resulting in these patients experiencing more trastuzumab-mediated ADCC. Although the mechanism through which this occurs is not fully appreciated, host immune cell factors and cancer cell characteristics could play a role. Although efforts were made to stain the CD14^+^ and CD56^+^ tumor infiltrated immune cells, satisfactory staining was not achieved (most likely due to the use of inappropriate antibodies). Salgado et al. of the Tumor Infiltrating Lymphocytes Working Group recently recommended that immunohistochemistry is not used to detect specific cellular subpopulations in clinical evaluation settings [24]. Therefore, we chose to determine the level of TI HER2 trogocytosis by evaluating the status of HER2 expression on tumor-infiltrated immune cells from HER2+ breast cancer patients who were due to be treated with trastuzumab-based PST. The patients were divided into 2 groups depending on the degree of trogocytosed-HER2^+^ tumor-infiltrated immune cells (Figure 4B); the patients who showed a high degree of HER2 expression on tumor-infiltrated immune cells (by TI HER2 trogocytosis) demonstrated a significantly greater probability of achieving a pCR with trastuzumab-based PST (Figure 4C). Previous studies have indicated that increased levels of tumor-infiltrated lymphocytes could be a predictive factor for PST response [25,26]. However, we identified 2 cases in which there was a high level of tumor-infiltrated lymphocytes, but low trogocytosis; neither of these patients achieved a pCR. As such, we believe the clinical application of our results could result in a more accurate prediction of HER2+ breast cancer patient’s response to PST (Figure 4B). Following the publication of a recent report which indicated that high HER2 protein and high *HER2* and *HER3* mRNA levels correlate with a better response to anti-HER2 antibody based treatment [27], it has been suggested that a high level of HER2 expression is necessary to achieve a good response to anti-HER2 therapy. Therefore, in considering the clinical importance of low HER2-trogocytosis, we suggest that HER2^+^ breast cancer patients whose tumor HER2 immunohistochemistry score is 3+ or 2+ might actually have a low quantity of HER2 protein. Patients whose tumors express low levels of the HER2 protein may exhibit a poor response to anti-HER2 antibody treatment due to low levels of HER2-trogocytosis in addition the effect that low levels of HER2 protein have on anti-HER2 therapy response. However, given the low number of patient samples in the current study, a larger patient population study is required in order to determine the true clinical significance of HER2-trogocytosis.

## Conclusions

TME interactions between immune cells, trastuzumab, and cancer cells are important when considering the anti-HER2 antibody treatment of HER2+ breast cancer patients. We have shown that immune cell trogocytosis prior to trastuzumab treatment may correlate with achieving a pCR with trastuzumab-based PST. Further evaluation is required to establish whether the response to HER2-targeted antibody therapy in HER2+ breast cancer patients can be determined by trogocytosis analysis, but we believe that future translational research to evaluate the clinical impact of trogocytosis in a cohort of anti-HER2 treatment trials is warranted.

## Additional files

Additional file 1: Figure S1A-B. There was no significant increase in HER2 expression on CD3^+^ or CD19^+^ cells in trogocytosis assays. The trogocytosis assay was performed with an E:T cell ratio of 10:1 and various concentrations of trastuzumab (H: H0, without trastuzumab; H1, 1 μg/mL of trastuzumab). HER2 positivity represents the uptake of HER2 onto CD14^+^, CD56^+^, CD3^+^, and CD19^+^ effector cells. **Figure S1B.** HER2^+^/CD14^+^ cells and HER2^+^/CD56^+^ cells have greater CD107a expression. Representative flow cytometry scatterplots of CD107a expression on HER2^+^/CD14^+^ vs HER2^−^/CD14^+^ and HER2^+^/CD56^+^ vs HER2^−^/CD56^+^ cells are shown.
Additional file 2: Figure S2. Expression of HER2 on tumor-infiltrated CD14^+^ and CD56^+^ cells in a HER2+ breast cancer patient treated with trastuzumab. Tumors from a trastuzumab treated HER2+ breast cancer patient and a HER2^−^ luminal type breast cancer patient were enzymatically dissociated to obtain cell suspensions which were subjected to flow cytometry in parallel with the patient’s PBMCs. The cells were stained with FITC-CD14, PE-CD56, and APC-HER2 antibodies. A CD14^+^ cells in patient PBMCs were gated against HER2 expression (APC; top 2 panels). CD14^+^ cells from dissociated tumors (bottom left panel, yellow circle) were gated against HER2 expression (APC) in both HER2+ and luminal type tumors (bottom right 2 panels). B CD56^+^ cells in patient PBMCs were gated against HER2 expression (APC; top 2 panels). CD56^+^ cells from dissociated tumors (bottom left panel, yellow circle) were gated against HER2 expression (APC) in both HER2+ and luminal type tumors (bottom right 2 panels). 
Additional file 3: Figure S3. Trastuzumab-mediated trogocytosis in autologous tumor cells and PBMCs from a HER2^+^ breast cancer patient. The tumor sample from the trastuzumab-untreated HER2+ breast cancer patient was enzymatically dissociated and the obtained cell suspension and patient’s autologous PBMCs were subjected to a trogocytosis assay (E:T cell ratio = 10:1; H0, without trastuzumab; H1, trastuzumab 1 μg/mL). A The expression of HER2 on CD56^+^, CD14^+^, and tumor cells in H0 and H1 treated samples is shown. In the CD56^+^ and CD14^+^ cell plots, the solid line represents HER2 staining and the dashed line represents isotype control staining. B Trastuzumab independent (TI) trogocytosis in autologous tumor cells and PBMCs. The TI trogocytosis assay was performed using dissociated HER2+ tumor cells and the patient’s autologous PBMCs with an E:T cell ratio of 1:1, 10:1, 100:1, and 1000:1 without trastuzumab treatment. The level of HER2 expression in the trogocytosed cells is shown as the percent of HER2^+^ cells in each condition. The figures show results from a single patient experiment.

## Abbreviations

HER2: Human epidermal growth factor receptor 2
PST: Primary systemic therapy
pCR: Pathological complete response
ADCC: Antibody-dependent cellular cytotoxicity
PBMC: Peripheral blood mononuclear cell
IC: Immune complex
FcγR: Fcγ receptor
NK: Natural killer
TME: Tumor microenvironment
E: Effector
T: Target
TD: Trastuzumab-dependent
TI: Trastuzumab-independent
ECD: Extracellular domain
HLA: Human leukocyte antigen
MHC: Major histocompatibility complex
