# Supplementary figures and images for: Trogocytosis-mediated expression of HER2 on immune cells may be associated with a pathological complete response to trastuzumab-based primary systemic therapy in HER2-overexpressing breast cancer patients
Source: BMC Cancer. 2015 Feb 6;15:39. doi: 10.1186/s12885-015-1041-3 (PMC4329225; doi:10.1186/s12885-015-1041-3)

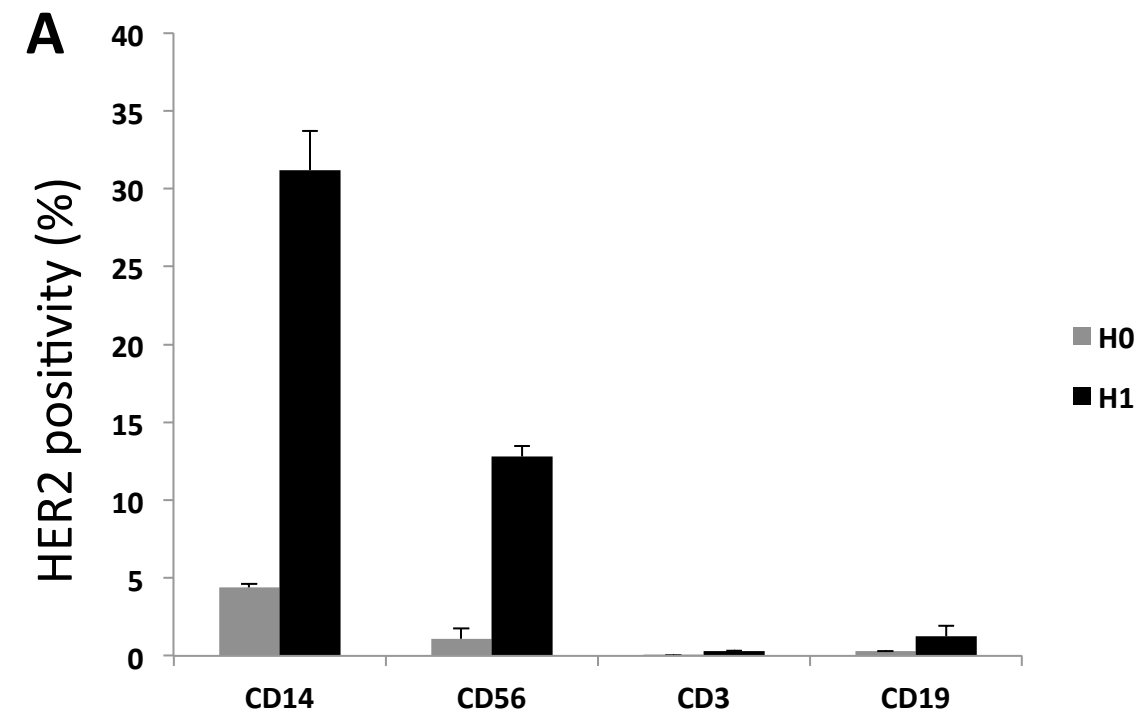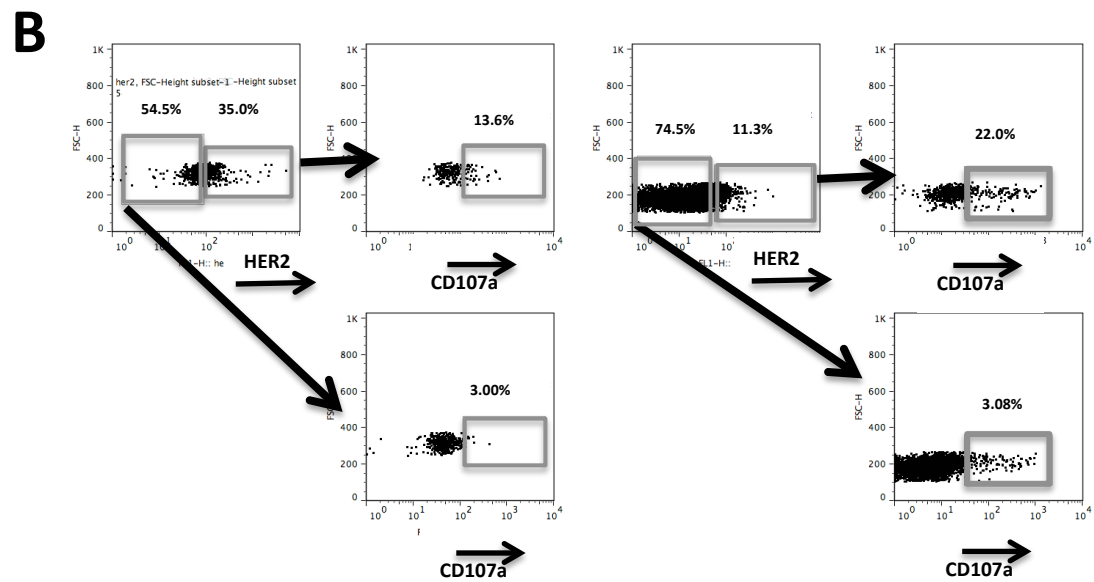

Supplement: Additional file 1: Figure S1A-B. — There was no significant increase in HER2 expression on CD3+ or CD19+ cells in trogocytosis assays. The trogocytosis assay was performed with an E:T cell ratio of 10:1 and various concentrations of trastuzumab (H: H0, without trastuzumab; H1, 1 μg/mL of trastuzumab). HER2 positivity represents the uptake of HER2 onto CD14+, CD56+, CD3+, and CD19+ effector cells. Figure S1B. HER2+/CD14+ cells and HER2+/CD56+ cells have greater CD107a expression. Representative flow cytometry scatterplots of CD107a expression on HER2+/CD14+ vs HER2−/CD14+ and HER2+/CD56+ vs HER2−/CD56+ cells are shown. [file 12885_2015_1041_MOESM1_ESM.pdf]

**A**

**CD14**

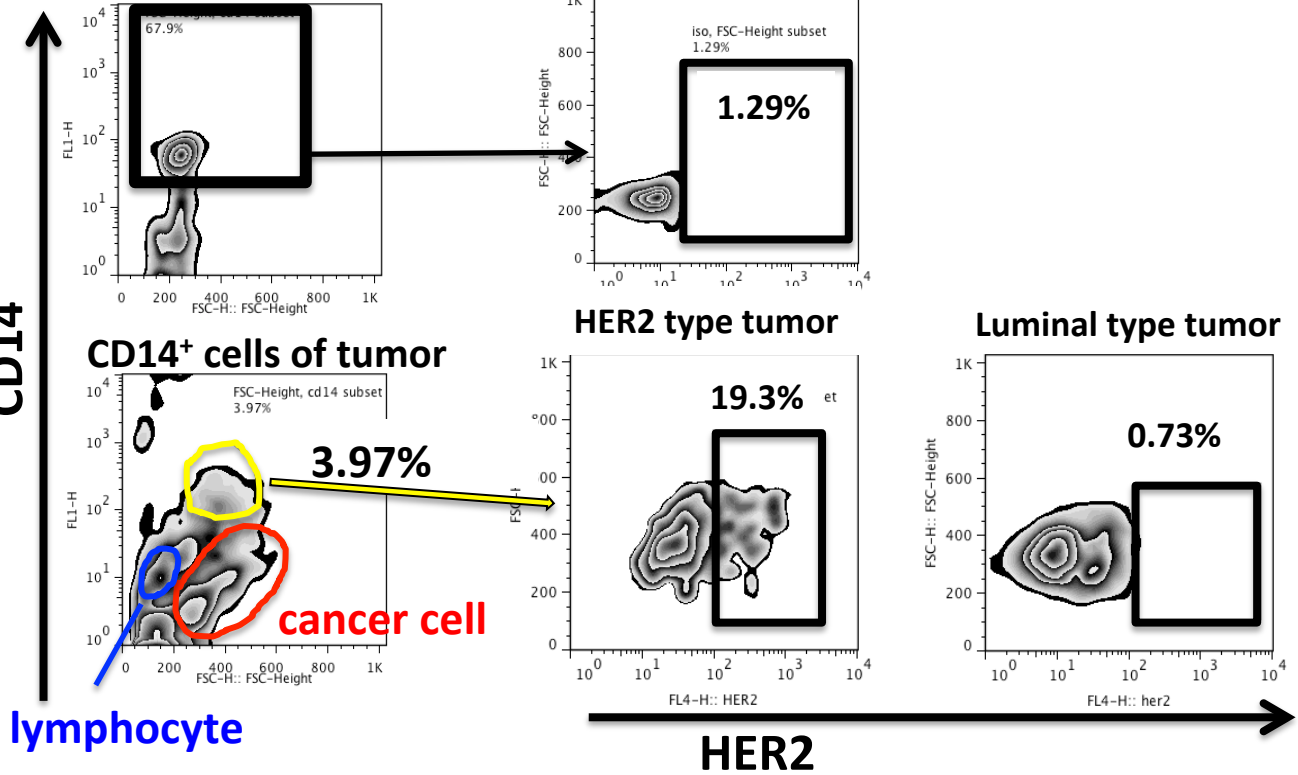

**B**

**CD56**

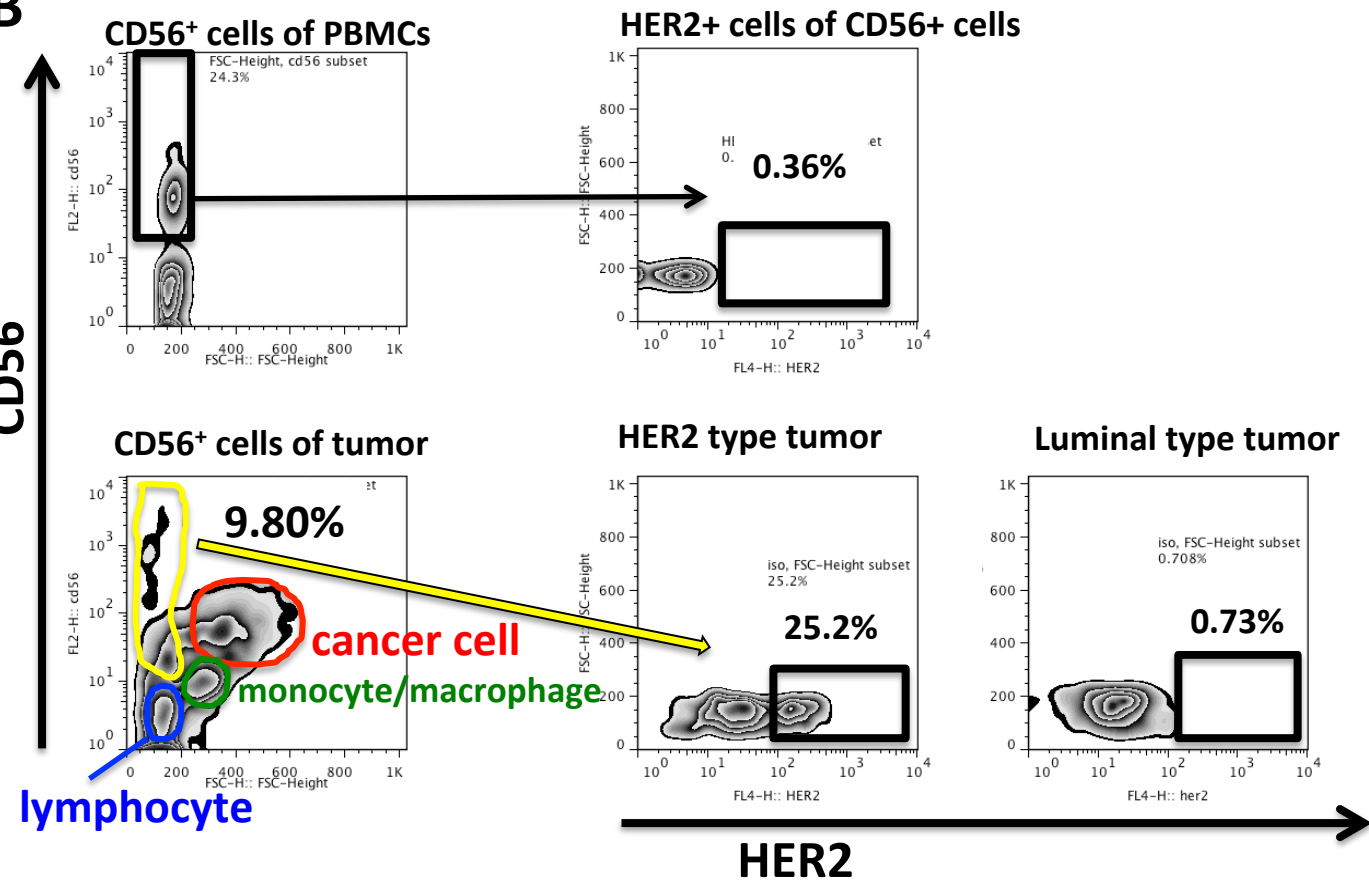

Supplement: Additional file 2: Figure S2. — Expression of HER2 on tumor-infiltrated CD14+ and CD56+ cells in a HER2+ breast cancer patient treated with trastuzumab. Tumors from a trastuzumab treated HER2+ breast cancer patient and a HER2− luminal type breast cancer patient were enzymatically dissociated to obtain cell suspensions which were subjected to flow cytometry in parallel with the patient’s PBMCs. The cells were stained with FITC-CD14, PE-CD56, and APC-HER2 antibodies. A CD14+ cells in patient PBMCs were gated against HER2 expression (APC; top 2 panels). CD14+ cells from dissociated tumors (bottom left panel, yellow circle) were gated against HER2 expression (APC) in both HER2+ and luminal type tumors (bottom right 2 panels). B CD56+ cells in patient PBMCs were gated against HER2 expression (APC; top 2 panels). CD56+ cells from dissociated tumors (bottom left panel, yellow circle) were gated against HER2 expression (APC) in both HER2+ and luminal type tumors (bottom right 2 panels). [file 12885_2015_1041_MOESM2_ESM.pdf]
