# Supplementary material for: Trogocytosis-mediated expression of HER2 on immune cells may be associated with a pathological complete response to trastuzumab-based primary systemic therapy in HER2-overexpressing breast cancer patients
Source: BMC Cancer. 2015 Feb 6;15:39. doi: 10.1186/s12885-015-1041-3 (PMC4329225; doi:10.1186/s12885-015-1041-3)
Supplement: Additional file 3: Figure S3. — Trastuzumab-mediated trogocytosis in autologous tumor cells and PBMCs from a HER2+ breast cancer patient. The tumor sample from the trastuzumab-untreated HER2+ breast cancer patient was enzymatically dissociated and the obtained cell suspension and patient’s autologous PBMCs were subjected to a trogocytosis assay (E:T cell ratio = 10:1; H0, without trastuzumab; H1, trastuzumab 1 μg/mL). A The expression of HER2 on CD56+, CD14+, and tumor cells in H0 and H1 treated samples is shown. In the CD56+ and CD14+ cell plots, the solid line represents HER2 staining and the dashed line represents isotype control staining. B Trastuzumab independent (TI) trogocytosis in autologous tumor cells and PBMCs. The TI trogocytosis assay was performed using dissociated HER2+ tumor cells and the patient’s autologous PBMCs with an E:T cell ratio of 1:1, 10:1, 100:1, and 1000:1 without trastuzumab treatment. The level of HER2 expression in the trogocytosed cells is shown as the percent of HER2+ cells in each condition. The figures show results from a single patient experiment. [file 12885_2015_1041_MOESM3_ESM.pdf]

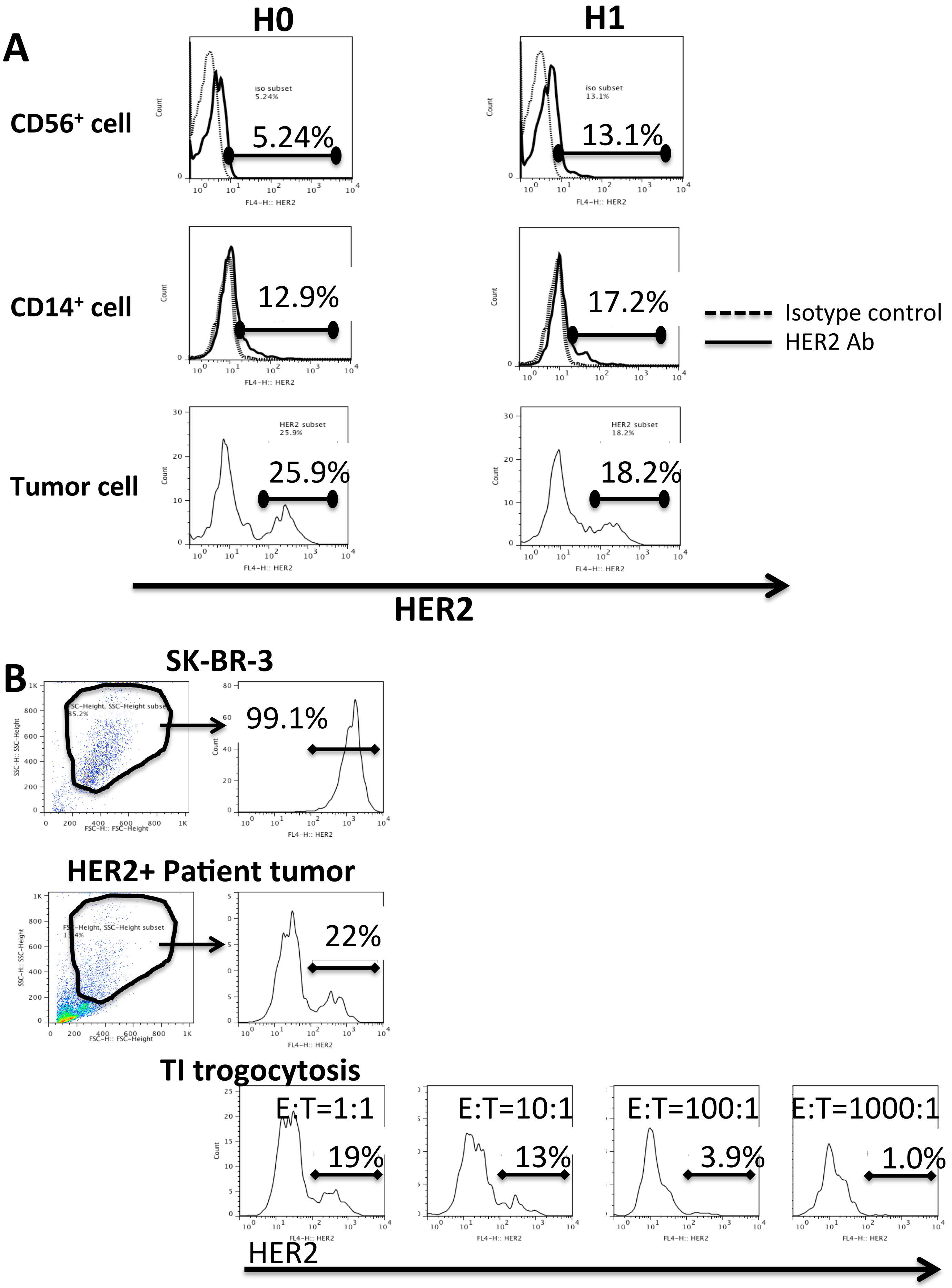

A
H0
H1
CD56+ cell
5.24%
13.1%
CD14+ cell
12.9%
17.2%
Isotype control
HER2 Ab
Tumor cell
25.9%
18.2%
HER2
B
SK-BR-3
99.1%
HER2+ Patient tumor
22%
TI trogocytosis
E:T=1:1
19%
E:T=10:1
13%
E:T=100:1
3.9%
E:T=1000:1
1.0%
HER2
